# On the Utility of Warm and Cold Bathing in the Treatment of Insanity

**Published:** 1851-02

**Authors:** 


### ECLECTIC DEPARTMENT,

### AND SPIRIT OF THE MEDICAL PERIODICAL PRESS

---

*On the Utility of Warm and Cold Bathing in the Treatment of Insanity.* By J. H. Worthington, M. D., Superintendent of the Friends' Asylum for the Insane, near Frankford, Pa. Read before the Association of Medical Superintendents of American Institutions for the Insane, convened in Boston, 6 mo. (June,) 1850.

It will, perhaps, be generally admitted, that the due performance of the function of cutaneous transpiration is an essential requisite to the healthy condition of the entire system. Labor, which has been the lot of the greater portion of the race since the first punishment was inflicted upon man by his Maker, seems to be the grand means appointed by nature to insure the healthy action of the skin. "In the sweat of thy face thou shalt eat bread till thou return to the ground," was the sentence pronounced against man for his first transgression, and it seems to have been imposed on him as a punishment from which there could be no escape, except at the risk of suffering and disease. To obviate the evil consequences which are likely to result from the neglect of this means of securing the healthy action of the skin, the resources of art are necessary; and among these there appears none capable of answering the purpose so beneficially as the use of the bath, under one or other of its different forms. With the advancement of luxury and refinement, the experience of all ages has proved the necessity of this means of preserving health; and the ruins of the public baths of the Romans, and other nations of antiquity, are evidences, yet remaining, of the estimation in which a healthy skin was held in those early periods.

The insane, either from physical inability, or from the want of that degree of reason which is necessary in the performance of most kinds of manual labor, as a class, must be deprived of all the benefits which accrue from this source; and, perhaps, from this cause, in great part, are subject to those chronic diseases which prove fatal to so large a proportion of the incurable insane.

The altered condition of the cutaneous surface is noticed by most writers on the subject of insanity. Almost all speak of coldness of the extremities and want of free circulation in the capillary system, in some forms of the disease. Diminution of cutaneous transpiration is manifested in many of the insane by a dry and harsh state and altered appearance of the surface, and a change in its quality is evident in the odor which is often given off by the bodies of the insane. Insanity, when it has become chronic, is apt to be accompanied by cutaneous disorders of different kinds, which are often very difficult to eradicate. Esquirol lays particular stress on the connection between insanity and eruptive diseases, and thinks it one which deserves attention. He speaks of the skin, in some cases, being in a state of erethism which is very remarkable, and of a renewed flow of the perspiration being followed by a resolution of the disease. In the affection called "pelagra," a diseased condition of cutaneous surface, sometimes accompanied with disorder of the digestive functions, ends in the gradual impairment of the intellect and senses, with a strong tendency to suicide.

The effects of the sudden suppression of perspiration by the application of cold, have long been observed under the form of fevers and dangerous inflammatory affections of dtfferent internal organs. Experiments upon the inferior animals, by coating their bodies with an impervious layer of varnish, demonstrate the injurious and violent effects produced by the repulsion into the circulation of the effete matters which it is the function of the skin to eliminate. A French physician, Fourcault, mentions the instance of a child, who was covered with gold leaf at the coronation of one of the Roman pontiffs, to represent the golden age which was to be revived by the reign of the new sovereign, who fell a victim to this act of presumption. The same author coated the skins of several Guinea pigs with gold and silver leaf, or tin-foil, so as completely to prevent the escape of the perspiration, and they all died in a short time with every appearance of asphyxia. When a smaller extent of surface was covered, the effects were less violent, and they consisted generally of a modification of some internal secretion. In some of the experiments a profuse discharge was produced from the nostrils, in others, diarrhœa ensued, and the mucous membranea, in either case, on examination, were found presenting the appearance of intense inflammation.

When these striking effects are seen to be produced by the sudden, and more or less complete suppression of the perspiration, it might be expected that in a less degree, but continued through longer periods of time, its detrimental effects upon health, though less cognizable, would be none the less real. We must look to classes of society and communities, whose habits and modes of living are such as to favor the habitual diminution of cutaneous transpiration, for evidence of the prejudicial influence exerted thereby upon health, as well as for the nature of the morbid changes it produces. The occupants of dark, damp, and ill-ventilated dwellings, operatives in manufactories, inmates of large alms-houses, convicts in prisons and penitentiaries, &c., have been especially noted for their unhealthiness, and the prevalence among them of scrofula, phthisis, &c., has generally been attributed to the influence of vitiated air upon the function of hæmatosis, and of insufficient food, combined with want of exercise upon that of nutrition. The observations of M. Fourcault, however, without denying to these causes a degree of influence in the production of the diseases named, seem to point more especially to derangement of the excretory functions of the skin as their origin or primary cause. This author concludes upon examination of all the circumstances attending the mode of life of the different classes of people enumerated above, that their unhealthiness is not so much owing to their being ill-fed and breathing a vitiated atmosphere, as to their habits of bodily inactivity, or to confinement to occupations which afford only a small amount of exercise. These views are sustained by the reports of the British Registrar General, and by M. Villirme, of France, who concur in the statement that by far the greater number of cases of phthisis, scrofula, &c., occurring among persons engaged in factory labor are presented by workmen whose occupation requires them to remain steadily in one position, and in whom, consequently, elimination by the skin cannot be assisted by exercise. The silk weavers of Lyons, and other towns in the south of France, are thus situated, and an immense proportion of the mortality among them is caused by consumption. In other towns where the manufacturing of cloth is carried on, which requires considerable activity and strength, consumption is much less frequent.

It is scarcely necessary to refer to the work* of M. Fourcault for further evidence of the greater liability to consumption and scrofula among persons pursuing sedentary occupations, and among the wealthy, who lead indolent and luxurious lives. Indeed, the prevalence of these diseases among the latter class shows this liability does exist, even when the other circumstances, which are thought to be most favorable to their production, are entirely wanting. The effect of a life of labor and hardship in preventing the development of tubercular disease, in persons predisposed to it, is well known; and perhaps we cannot explain this exemption more satisfactorily, than by referring it to the beneficial influence upon all the other functions of the natural secretion of perspiration which is thus insured. The inference, that diminution in the quantity of fluid perspired is a principal cause of the development of consumption, is materially strengthened by the fact of the great prevalence of the disease in countries having a moist and cool atmosphere, as England, where the mortality therefrom is as high as one-third of the whole number of deaths. It has also been remarked, that individuals predisposed to phthisis may reside for a long time in a warm climate and continue to enjoy good health, but on removing to a colder one are exceedingly liable to have the disease developed. A large proportion of animals exhibited in menageries, natives of warm regions, die of tubercular diseases, and in these cases it would, perhaps, be difficult to account for the result in any other way, than by attributing it to the diminution or suppression of the insensible perspiration, caused by the colder atmosphere.

The mode in which derangement of the function of the skin acts in producing disease, may be understood on the physiological law of vicarious secretion. The mucous membranes and the skin present many points of resemblance, both of structure and function, and the former may, therefore, with good reason, be supposed to supply the place of the latter, when, from any cause, it is rendered unfit for performing its functions. On this hypothesis we would consider the increased secretion from the mucous membrane of the nostrils in ordinary catarrh merely to have taken the place of the natural cutaneous excretion, and in bronchitis the irritation of the mucous membrane would be produced by the effort to throw off from the system, through this channel, the retained matter of the perspiration. Diarrhœa, and the various forms and grades of enteritis, are caused by sudden cooling of the surface of the body, and their production might be accounted for in the same manner.

However we may attempt to explain the occurrence of inflammatory affections of the lungs, bowels, and kidneys, consequent upon exposure to cold, the belief that they are intimately connected with suppressed perspiration is warranted by the trials of M. Fourcault on animals, as well as sanctioned by common experience. It is not, however, with acute diseases

---

* Causes generales des maladies chroniques, specialement de la phthisie pulmonare. Vide Am. Journal Med. Science, Oct., 1845.

of these organs that we have to contend in the treatment of the insane. Their cutaneous functions are seldom interrupted by sudden vicissitudes of temperature. They become deranged in a more gradual manner, and hence those organs which sompathize more directly with the skin,—which are most liable to disease on the sudden suppression of its functions,—suffer more slowly, and become afflicted with various chronic disorders. It does not appear to be an unwarrantable conclusion, that in this way originate a great part of the consumption, the chronic diarrhœa and dysentery, the marasmus and dropsies, which cause such a large proportion of the deaths in lunatic Asylums.

If the foregoing views respecting the importance of the functions of the skin, and their liability to derangement in the various forms of insanity, be correct, the necessity is obvious of attending to these functions, and of preserving them in as healthy a condition as possible, by the employment of the only means to which we can conveniently resort.

The forms of bathing which are most generally applicable in the treatment of insanity, are, the warm bath averaging from 92 to 98 deg. Fahrenheit, and the cold bath, at an average temperature of 52 deg. Fahrenheit. Independent of their direct influence upon the surface of the skin, tending to remove obstructions to the free performance of its functions, and thus promoting a healthy action of the internal organs, the beneficial effects of warm and cold bathing, in many cases of mental disorder, are such as can scarcely be obtained by any other means. The days in which it was considered necessary to treat every case of disordered cerebral circulation by a resort to depletory measures have happily passed by, and the end which was sought to be attained thereby, may be answered, in many cases of insanity, by other means, including the use of the warm bath. The diseased action of the brain, even in the early periods of insanity, is rarely of that kind which is attended with high arterial excitement. It is, at least, many degrees removed from true inflammation, whose natural termination is effusion of lymph or pus, and which requires the adoption of the most vigorous measures for the prevention of fatal or permanent disorganizations. Produced in most cases by causes which tend gradually to lower the tone of the system, experience has shown the necessity of avoiding every means calculated still further to reduce the strength. It is, then, in a condition of diminished vital activity, that we have to apply our remedies, while the brain is, notwithstanding, suffering from a too great quantity of blood circulating through its vessels; other organs, perhaps, at the same time, receiving a deficient supply. Under these circumstances, the use of the warm bath is attended with peculiar advantages. It acts by inviting the circulating fluid to the surface, and filling the capillary system of vessels, by which means the vital fluid distributed to every part of the system is equalized, and the brain relieved of its surplus, without the evil consequences resulting which are to be feared from direct depletion. In cases which are attended with considerable nervous irritability and morbid wakefulness, the warm bath, taken at bed-time, will often prove serviceable by its soothing influence in allaying excitement, and disposing to sleep when narcotics would be inadmissible or fail of their usual effects. It may act here also, by remedying a slighter degree of unequal distribution of the circulating fluid, which often accompanies this condition of the nervous system. To many persons in ordinary health, travelling is apt to be productive of a certain degree of morbid irritation of the nervous system, attended with dryness of the skin, slight feverishness, and a defective performance of some of the secretory functions, for the removal of which the warm bath is one of the most effectual remedies. In most insane persons, these effects may be expected to be produced in a more marked degree by their journey to the Instiution where they are to undergo a course of treatment, and the bath, were there no other reasons for its employment, will therefore frequently be found serviceable, immediately after the admission of the patient. The effect of the warm bath is to soothe and refresh the whole system, and at the same time to render all its functions free and of more ready performance. It is, therefore, probable that it would be found highly beneficial in those cases of high maniacal excitement, which, from their being accompanied with almost entire want of sleep and obstinate refusal of nourishment, are apt to terminate in fatal exhaustion. In such cases, the functions of the skin, as well as those of the gastro-enteric mucous membrane, are greatly perverted, the surface of the body and the extremities being in most cases cool, and either moist or excessively dry and harsh, and presenting to the eye an earthy or dusky appearance.

In no form of insanity has the diseased condition of the cutaneous surface been more frequently noticed than in melancholia. Esquirol speaks of the skin as being brown, blackish, dry and scaly, while in no form of insanity is there so strong a tendency to those chronic affections which, as has been attempted to be shown, are probably dependant on disordered cutaneous function. Of 196 deaths of patients affected with melancholia, who came under the care of this distinguished author and physician, 62 were of phthisis, 32 were of chronic affections of the bowels, and 24 were of marasmus. If there be any such connection as has been suggested, between the supposed cutaneous function on one hand, and the diseased thoracic and abdominal viscera on the other, it is evident that any means capable of remedying the former would be the most effectual in preventing the latter. In this form of insanity, therefore, it is probable that the frequent use of the warm bath would be found highly beneficial in its tendency to prevent the development of those diseases which prove fatal to so large a proportion of melancholiacs.

In a large number of insane persons there is a striking defect of the function of capillary circulation, in connection with a torpid condition of the entire system. In these cases the cutaneous surface, especially on the extremities, is constantly cool, sometimes moist and clammy. The skin is discolored, and if the blood circulating in it be temporarily removed by pressure, it is a long time in resuming its original color. There is a defective performance of the digestive functions, the bowels are sluggish in their action, and the brain seems to suffer from a deficient supply of arterialized blood. This condition of the physical functions is frequently accompanied by great mental apathy, from which it is almost impossible to arouse the patients, and many of them are addicted to depraved personal habits, which tend to their deterioration of body and mind. In these cases the cold bath acts most beneficially, by calling into exercise the conservative powers of the system, and by the reaction thus produced tending directly not only to quicken the circulation in the capillary vessels, but to promote the vital activity of the whole system. In many cases of hypochondriasis and excessive nervous irritability, after the disordered condition of the abdominal viscera has been in some measure corrected, the cold bath, by its general tonic effects and by restoring a healthy condition of the cutaneous functions, will often be found advantageous.

The cold douche, as a form of local bathing, has been too much employed in the treatment of the insane to be passed by without notice. In many cases of high cerebral excitement, the application of a stream of cold water falling from a height upon the head, has been found to be a valuable auxiliary to other necessary treatment; and when there is an unequal distribution of the circulating fluid, it will, especially in connection with the warm bath or pediluvium, often prove of service in insanity. Where there is considerable vigor of the general circulation, particularly in young persons, the frequent use of the douche is productive of a feeling of freshness and comfort which is very grateful, and which may be indicative of improvement in the state of the cerebral circulation. It is beyond the province of this report to enter further upon the subject of the so called moral treatment of insanity by means of the douche, further than to remark that its general use, as well as that of the shower bath, as a means of repression or punishment would probably be destructive, by the feelings of fear or dislike which it would inspire, of all the benefits likely to be derived from their use as therapeutic agents.

The foregoing article is from the American Journal of Insanity. Some remarks on the construction of baths are omitted.—Editor Buffalo Medical Journal.

